# Monitoring maternal, newborn, and child health interventions using lot quality assurance sampling in Sokoto State of northern Nigeria

**DOI:** 10.3402/gha.v8.27526

**Published:** 2015-10-09

**Authors:** Dele Abegunde, Nosa Orobaton, Kamil Shoretire, Mohammed Ibrahim, Zainab Mohammed, Jumare Abdulazeez, Ringpon Gwamzhi, Akeem Ganiyu

**Affiliations:** 1 United States Agency for International Development – John Snow Inc. Research and Training, Inc. – Targeted States High Impact Project Nigeria; 2 Jhpeigo – Targeted States High Impact Project Nigeria, Bauchi, Nigeria

**Keywords:** monitoring and evaluation, maternal, newborn and child health, lot quality assurance sampling, Nigeria

## Abstract

**Background:**

Maternal mortality ratio and infant mortality rate are as high as 1,576 per 100,000 live births and 78 per 1,000 live births, respectively, in Nigeria's northwestern region, where Sokoto State is located. Using applicable monitoring indicators for tracking progress in the UN/WHO framework on continuum of maternal, newborn, and child health care, this study evaluated the progress of Sokoto toward achieving the Millennium Development Goals (MDGs) 4 and 5 by December 2015. The changes in outcomes in 2012–2013 associated with maternal and child health interventions were assessed.

**Design:**

We used baseline and follow-up lot quality assurance sampling (LQAS) data obtained in 2012 and 2013, respectively. In each of the surveys, data were obtained from 437 households sampled from 19 LQAS locations in each of the 23 local government areas (LGAs). The composite state-level coverage estimates of the respective indicators were aggregated from estimated LGA coverage estimates.

**Results:**

None of the nine indicators associated with the continuum of maternal, neonatal, and child care satisfied the recommended 90% coverage target for achieving MDGs 4 and 5. Similarly, the average state coverage estimates were lower than national coverage estimates. Marginal improvements in coverage were obtained in the demand for family planning satisfied, antenatal care visits, postnatal care for mothers, and exclusive breast-feeding. Antibiotic treatment for acute pneumonia increased significantly by 12.8 percentage points. The majority of the LGAs were classifiable as low-performing, high-priority areas for intensified program intervention.

**Conclusions:**

Despite the limited time left in the countdown to December 2015, Sokoto State, Nigeria, is not on track to achieving the MDG 90% coverage of indicators tied to the continuum of maternal and child care, to reduce maternal and childhood mortality by a third by 2015. Targeted health system investments at the primary care level remain a priority, for intensive program scale-up to accelerate impact.

Counting down to December 2015 (1), Nigeria, with its 160 million population and approximately 20% of Africa's population, is behind in Millennium Development Goals (MDGs) 4 (reduce child mortality) and 5 (improve maternal health) targets (1, 2). Sokoto State in northwest Nigeria with a maternal mortality ratio (MMR) of over 1,540/100,000 is three times the national ratio (3, 4). About 11.5% of births in this region and 4.7% of births in Sokoto State occurred in health facilities compared to 35% nationally (4). The northern region of the country including Sokoto State, accounts for 94% of all unassisted deliveries with no one present (2, 3). Available statistics show neonatal mortality rate to be 37 deaths per 1,000 live births in 2013 (4, 5); infant mortality rate (IMR) was 78 per 1,000 live births in 2013, and total under-five death rate was 185 per 1,000 live births (4, 5). Demand for family planning (FP) satisfied was 43%, 45% of pregnant women attended at least four antenatal clinic visits during pregnancy, 39% of deliveries were supervised by a skilled birth attendant, and 13% of mothers practiced exclusive breast-feeding. Diphtheria–Pertussis–Tetanus (DPT3) coverage rate was 47%, and 23% of under-five children received antibiotic treatment for pneumonia (1, 3). Nigeria is estimated to have achieved only 0.9% average annual rate of reduction in MMR and 3.8% reduction in under-five mortality rate between 2000 and 2011 (1).

Since 2009, the United States Agency for International Development (USAID) has supported the Targeted States High Impact Project (TSHIP), managed by John Snow Incorporated Research & Training Institute, Inc., to improve maternal, newborn, and under-5 children's health status and survival. The intervention program focused on improving and strengthening linkages across the continuum of maternal, neonatal, and child health care from prepregnancy, through pregnancy, childbirth, and the early days and years of life (1) for women and under-5 children. Technical assistance was provided to strengthen the state government capacity for delivery and promotion of high-impact integrated management of maternal, neonatal, and child health (MNCH)/FP/reproductive health interventions at primary healthcare centers. The project enhanced health providers’ capacity to deliver and promote the use of high-impact interventions and to enhance the role of households and communities in health promotion. In addition to strengthening the existing referral system, interventions also targeted improved resource allocation and policy implementation at the state and local government levels. A strong component of the project was the establishment and training of community structures: Ward development committees and more than 3,000 community-based health volunteers (CBHV) provided linkages between the communities and the public primary care facilities, in addition to operating as demand creators. The CBHVs constantly identified women who became pregnant, and provided home visitations, and some health education and motivation to attend antenatal clinics, in addition to other health-promoting activities such as supporting immunization of the children. The United Nations Commission on Information and Accountability for Women's and Children's Health selected indicators to monitor progress across the continuum of maternal and child health care, in the countdown to 2015 (1). They include demand for FP satisfied, at least four antenatal care (ANC) visits, skilled attendant at birth, postnatal care for mothers and babies within 2 days of birth, exclusive breast-feeding for first 6 months of life, three doses of combined DTP3 immunization coverage, and antibiotic treatment for pneumonia. These indicators have been used to profile countries’ efforts toward the achievement of the MDGs 4 and 5 and to monitor and evaluate gains in maternal and child health programs and interventions (1) (Table 1).

**Table 1:** National averages, estimated coverage across the continuum of maternal, neonatal, and child health care indicators: 2012 and 2013

				Estimated average coverage		
				
Continuum of care	Indicators	National estimates – DHS 2008 (3)/countdown (1) (%)	National estimates DHS 2013 (4)	2012% (±)	2013% (±)	Difference: 2013–2012
Prepregnancy	Demand for FP satisfied	15	15	7.2 (0.5)	7.69 (0.6)	0.49
Pregnancy	Antenatal care (4 + visits)	45	61^a^	17.8 (1.23)	18.8 (1.8)	1.0
Birth	Supervised birth attendant	39 (35)	38	17.6 (1.1)	16.3 (1.0)	−1.3
Postnatal care	Postnatal care for mothers	–	–	6.16 (0.5)	10.11 (0.5)	3.95
	Postnatal care for baby	–	–	3.06 (0.02)	1.69 (0.2)	−1.37
Childhood	Exclusive breast-feeding: under-6-month old	13	17	63.15 (2.4)	65.53 (2.5)	2.38
	DPT3 vaccine	35	38	2.86 (0.2)	1.87 (0.2)	−0.99
	Antibiotics for pneumonia: under-5-year old	23	29	13.5 (0.78)	26.06 (1.6)	12.6

a Proportion of women who reported consulting a skilled health provider – a doctor, nurse, midwife, or auxiliary midwife – at least once for antenatal care for the most recent birth in the five-year period before the survey. DHS, demographic health survey; FP, family planning.

## Objective

The objective was to use the continuum of care framework to evaluate the progress of Sokoto State toward achieving the MDGs 4 and 5 by 2015 and to assess the impact of interventions implemented between 2012 and 2013.

## Methods

USAID/TSHIP program adopted the lot quality assurance sampling (LQAS) methodology to assist Sokoto State government to monitor its programs and interventions (6). The project conducted annual surveys as its monitoring strategy. LQAS is a relatively rapid (7), statistically sound 8, and inexpensive small sample alternative to traditional population-type surveys which is gaining popularity for quick and frequent evaluation of interventions and programs in the field of population health (6, 8–17). It is most useful for determining whether an intervention, a program, or a ‘supervision area (SA)’ met set targets or coverage thresholds (18, 19). Depending on performance, intervention programs or SAs are then classified into high- or low-priority areas. Estimates from the SAs are aggregated to determine the coverage in the catchment areas (9, 10).

Using the LQAS technique (6), a baseline evaluation study was carried out in 2012. A follow-up study was conducted in 2013 to monitor the impact of interventions and progress toward the MDG in Sokoto State. Data were collected through a rigorous multistage random sampling of 19 LQAS locations (villages) in each of the 23 local government areas (LGAs) (SA or Lots). In each year, 19 households were randomly selected from the sampled villages (totaling 437 households, combining to 874 in the 2 years) in the state catchment area – using probability proportionate to population size – considered a ‘self-weighing’ sampling design (20, 21). A household was defined as groups of persons who ate from the same cooking pot. A sample of 19 respondents from each of the sampled locations in LGAs (SA) provided acceptable levels of statistical error (90–95% confidence level, classification errors; α≤5%, and β≤20%) (8, 22) to inform decisions (9). Larger samples have the same statistical precision and are costlier (23). The LGA coverage estimates of the respective indicators were aggregated to the state's coverage estimates. Data collection followed an intensive 9-day training of data collectors and field supervisors drawn from the state's health workforce. Sampling frame of list of settlements was developed for each SA from which households were sampled.

Data were obtained using a set of well-tested questionnaires that included indicators applicable to the continuum of maternal and child health care from prepregnancy, through birth, to the immediate postpartum period. Data collection used a standardized, quality controlled, and validation process. Ethical approval was obtained from Sokoto State Health Research Ethics Committee. Signed, informed consent was obtained from respondents prior to interviews.

Data entry was done using EPI-INFO software. Microsoft Excel and STATA© statistical packages were used to obtain coverage estimates (defined as the proportion of individuals needing a service or intervention who actually received it) for each of these seven indicators. LGAs were classified as having acceptable levels if they achieved the MDG-recommended global average of 90% coverage benchmark, or unacceptable if they fell on or below the national coverage from Demographic Health Survey (DHS) 2013 (4), based on a statistically determined decision rule (*d*), adjusted to the sample size and a predetermined coverage threshold. The state (average) coverage was estimated upon which the LGAs’ coverage estimates were compared. The confidence intervals (CIs) of the estimates of the state average coverage were calculated using finite population correction factor technique (24). For programmatic purposes, an LGA was determined to be a high priority for programmatic efforts and resource reallocation if it performed below the acceptable benchmark and below the national average or low priority if the LGA had achieved the benchmark.

Sokoto State is located in the northwestern geopolitical zone of Nigeria with an estimated population of 5.7 million. It comprises 23 administrative LGAs in three (north, south, and central) senatorial zones. Sokoto State had a total fertility rate of 7.0 in 2013, among the highest in Nigeria. In 2013, only 1% of children below 12 months of age had received all basic immunizations in Sokoto State, compared to 23% at the national level in 2013 (4). Contraceptive use rate in 2013 among married women in the northwest was 4% and 1% in Sokoto State (4).

## Results

**Fig. 1:**
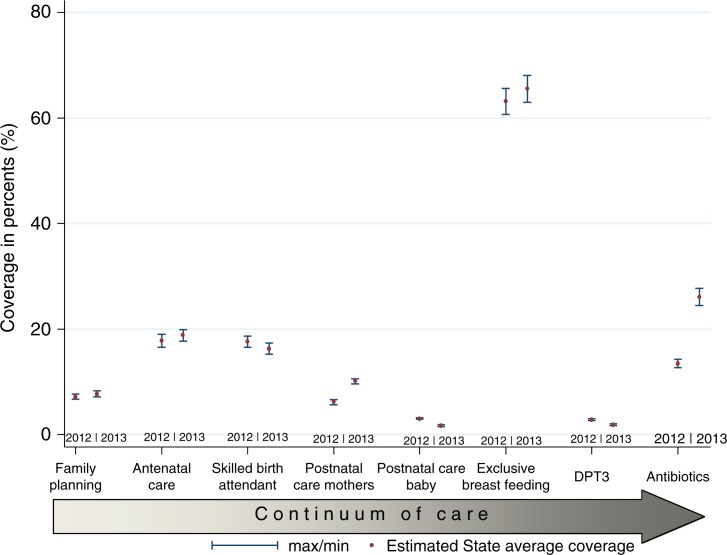
Coverage along the continuum of maternal newborn and child health care, 2012 and 2013.

As shown in Table 1, the estimated average coverages of all the indicators in the year 2012 (except for exclusive breast-feeding) were much lower than the MDG-recommended 90% coverage and the national average for the same year (Fig. 1). The estimated demand for FP satisfied of 7.2 (95% CI:±0.5%) was much lower than the 2008 and 2013 national averages of 15%. Only 17.8% (95% CI:±1.23%) of pregnant women had at least four ANC visits during their pregnancies compared to 2008 national average of 45% and 2013 national average of 61%. About 17.6% (95% CI:±1.1%) of deliveries were supervised by a trained attendant in 2012. Only 6.16% (95% CI:±0.5%) of women had at least one visit by the fourth day after delivery and 3.06% (95% CI:±0.02%) of the newborns were visited within the first 4 days of birth. Only 2.86% (95% CI:±0.2%) of children have had DPT3 vaccine and 73% (95% CI:±2.5%) of children with suspected pneumonia significantly had antibiotic treatment. About 63% (95% CI:±2.4%) of children less than 6 months old were breast-fed exclusively 24 h prior to the day of survey.

In contrast, in 2013 the demand for FP increased by less than one percentage point from 7.2% in 2012 to 7.69% (95% CI:±0.6%). In the same period, ANC coverage increased by one percentage point from 17.8 (95% CI:±1.23%) in 2012 to 18.8% (95% CI:±1.8%). The proportion of women delivered by skilled attendants dropped by 1.3 percentage points from 17.6% (95% CI:±1.1%) in 2012 to 16.3% (95% CI:±1.0%) in 2013. Postnatal care for mothers within 4 days increased by four percentage points to 10.11% (95% CI:±0.5%) in 2013 from 6.16% (95% CI:±0.5%) in 2012 and postpartum care for children 4 days postdelivery decreased by 1.37 percentage points to 1,7% (95% CI:±0.2%) in 2013 from 3.1% (95% CI:±0.02%) in 2012. Exclusive breast-feeding in the under-6-month-old children 24 h prior to the survey increased by 2.4 percentage points to 65.5% in 2013 from 63.15% (95% CI:±2.4%) in 2012. DPT3 vaccination coverage decreased by one percentage point from 2012 level of 2.86% (CI:±0.2%) to 1.87% (CI:±0.2%) in 2013. Coverage of antibiotic treatment for suspected acute pneumonia increased by 12.6 percentage points from 13.5% (CI:±0.78%) in 2012 to 26.06% (CI:±1.6%) in 2013. Of all the indicators, only access to antibiotic treatment for pneumonia, which increased by 12.6 percentage points, and postnatal care for mothers which increased by four percentage points were substantial and statistically significant (Table 1, Fig. 1). The difference in the estimated coverage between 2012 and 2013 found in DPT3 (0.9 percentage point), and postnatal care for the newborn (1.37 percentage points) were statistically significant although they were minimal (Fig. 1).

**Fig. 2:**
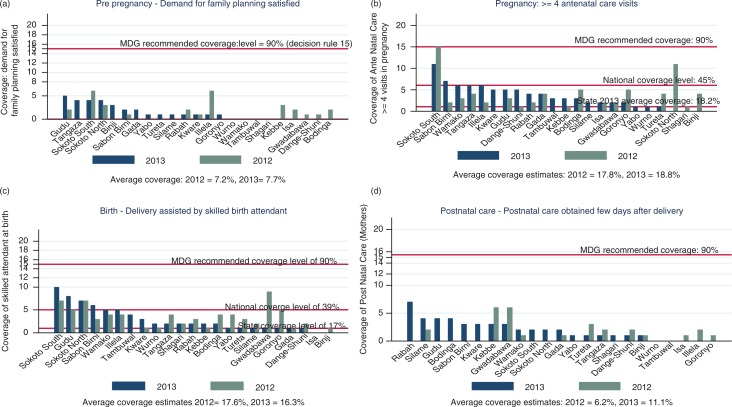
(a) Coverage of levels of demand for family planning, (b) antenatal care visits, (c) skilled attendant at delivery, and (d) postnatal care, by LGAs in 2012 and 2013.

**Fig. 3:**
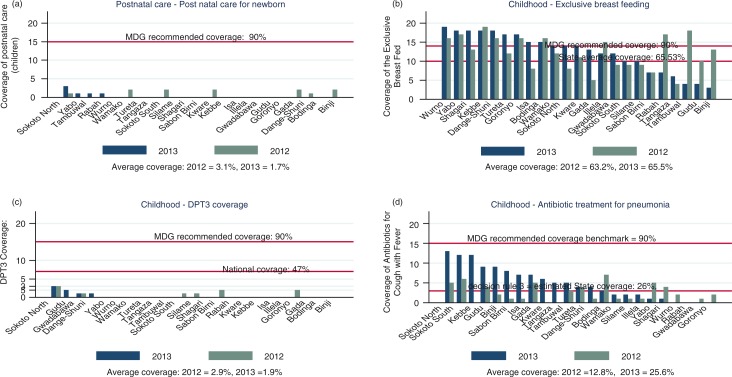
(a) Coverage of postnatal care for newborns, (b) exclusive breast-feedings, (c) DPT3 vaccination, and (d) antibiotic treatment for pneumonia, by LGAs in 2012 and 2013.

With respect to the performance at the LGA level, the demand for FP generally increased in 2013 over 2012 in all the LGAs except for three; Sokoto South, Rabah, and Ilela LGAs, where the demand fell to lower levels from 2012 to 2013 (Fig. 2a). All the LGAs were below the national coverage level of 43% in 2012 and in 2013. Many more LGAs increased in the demand for FP in 2013 from in the 2012 levels although almost all the increases were below the national average of 43%. Demand for FP was 0 in 2012 and 2013 in about 4 LGAs (Wurno, Wamako, Tambuwal, and Shagari) and 5 LGAS that had had some appreciable coverage in 2012, had zero coverage in 2013. Only 3 of the 23 LGAs achieved above the national average of 45% of pregnant women who had at least four ANC visits during their last pregnancy in 2012, one of which (Sokoto South), reached the 90% in the same year (Fig. 2b). Although most of the LGAs achieved higher coverage of antenatal visits in 2013 than in 2012, the majority was below the national average and some were below the estimated state-level coverage. Three LGAs, (Gwadabawa, Sokoto South, and Sokoto North), recorded a percentage of women whose deliveries were supervised by a skilled birth attendant higher than the national average of 39% in 2012. This increased to four LGAs in 2013 (Fig. 2c). The majority of the LGAs were below the national average and the estimated state average. Although the proportion of mothers who had had postnatal care within 4 days after delivery fell from 28% (95% CI:±2.22%) in 2012 to 10% (95% CI:±0.5%) in 2013, Fig. 2d shows that the number of LGAs which achieved any coverage in postnatal services increased from 12 in 2012 to 18 of the 23 LGAs in 2013. Nine LGAs that had had zero coverage in 2012 achieved marginally improved coverage in 2013. With respect to postnatal coverage for the newborn, Fig. 3a shows that whereas seven LGAs recorded some level of coverage in 2012, only four LGAs achieved any level of coverage in 2013. The level of coverage achieved in any LGA was so limited that it may partly explain the high IMR in Sokoto State and in the northwest region of Nigeria.

Overall, infant breast-feeding practices improved from 2012 to 2013 (Fig. 3b). Marginal improvement in coverage was achieved in the LGAs: 11 LGAs achieved the MDG-recommended level of 90% in 2012 and 2013 and 7 LGAs improved coverage to achieve either above the state average or above the MDG-recommended coverage although coverage dropped in seven LGAs, in 2013 from 2012 levels. As shown in Fig. 3c, six LGAs achieved at least a marginal (15%) DPT3 coverage in 2012. This reduced to four LGAs in 2013 and all were below the national average of 47%. The state (average) coverage was too small to be estimated within the statistical boundaries of LQAS decision rule table. Access to antibiotic treatment to treat acute pneumonia improved significantly in 2013. Coverage was above the state average of 26% in 13 of the 23 LGAs comprising the state (Fig. 3d).

## Discussion

We used data from LQAS surveys conducted as a monitoring tool to assess the impact of maternal and child health interventions in Sokoto State in northern Nigeria, between 2012 and 2013, in the context of the continuum of maternal and child care (1). Within the framework of the indicators for monitoring the continuum of maternal and child care (1), we evaluated the progress of Sokoto State toward achieving the MDGs 4 and 5 in the countdown to December 2015 (1). The results of this study were mixed with implications for future programming and intervention for reducing maternal and child deaths beyond December 2015. None of the eight indicators for measuring the continuum of maternal and childcare reached either the national coverage levels or the recommended 90% threshold for achieving the MDG 4 and 5 targets. The state (average) coverage estimates fell below the national coverage. The coverage of some indicators in some LGA worsened to zero. While the coverage of mothers whose deliveries were supervised by skilled birth attendants, postnatal care for newborns, and DPT3 vaccination worsened in 2013, satisfied demand for FP, antenatal and postnatal care visits, breast-feeding of infants, and antibiotic treatment for suspected cases of pneumonia marginally improved by a few percentage points. While access to antibiotic treatment increased by 12.6 percentage point, postnatal care, exclusive breast-feeding, ANC visits, and demand for FP improved by only 3.95, 2.38, 1.0, and 0.49 percentage points, respectively. The majority of the LGAs were classifiable as high-priority areas and therefore, were candidates for program intensification or reappraisal of the intervention strategies including the validity of prevailing assumptions. The progress toward the desired maternal and newborn health goals may be hampered with less likelihood of achieving the MDG targets, if these declines in the coverage both at the state and LGA levels persist.

Interplay of a multiplicity of factors may explain the observed low performance. Preprogram projections may have been underestimated because of the limited quality of data that may have been used for estimating preintervention coverage projections. Low-quality health data in sub-Saharan African countries including Nigeria are well-known to plague planning of program and intervention. Interventions may not have sufficiently circumvented barriers (especially sociocultural and socioeconomic barriers) to accessing health care for greater intervention effectiveness. Although additional resources have been injected into the health system in Nigerian states, its functionality remains constrained by many years of underfunding, worsened by inefficiency of allocation of funding (25, 26). Primary care in Nigeria experienced decades long funding neglect and infrastructural and human resource declines with devolution of authority to the LGA level (27–29). The gross underutilization of public healthcare facilities, particularly in northern regions of Nigeria, may have undermined the impact of interventions targeted at improving maternal and child health. Although data were unavailable to elucidate the implications for equity in access to continuum of care services and intervention, the low performance of the state in the coverage of most of the indicators of continuum of care was significant concern for the lower socioeconomic groups. These are often women and children in sub-Saharan Africa's context. Nigeria together with Chad, Somalia, Ethiopia, and Laos, have been identified as most inequitable countries for interventions in the continuum of care among the sub-Saharan countries (30). Community-based interventions were also found to be more equitably distributed than health-facility-based interventions.

It is noteworthy that the 1-year comparison window in this study may not be sufficiently sensitive to detect more subtle differences that have evolved. However, there were huge gaps between the coverage estimates on the one hand and national coverage levels and the recommended levels for achieving MDGs 4 and 5 on the other, for all of the indicators of the continuum of care. Achieving the desired coverage levels remains critical. Results of a previous study in Bauchi, a northeastern state, also used similar LQAS data and reported similar inter-LGA variations in the indicators of continuum of care although only modest improvements in these indicators were reported (31). Studies have used data from various other methodologies for similar exploration such as data from national surveys (32), a LIST model exploration (33, 34), and a health system evaluation framework developed by Countdown In-Depth Country Case Studies (34). These explorations have used data, which spanned longer periods than the surveys used in this study but with similar implications for the assessment of the continuum of care indicators as in this study. The limitations in the use of 1-year interval LQAS surveys therefore, do not diminish the findings from this exploration that Sokoto State and indeed Nigeria are constrained in their performance in MDGs 4 and 5.

## Conclusions

Despite the limited time left in the countdown to December 2015, the coverage levels of the indicators for measuring the continuum of maternal and childcare were critically short of the recommended levels for achieving MDGs 4 and 5. Coverage estimates were also below the national average levels. Intensive scale-up of programs and interventions would be needed to accelerate, consolidate, and sustain the modest achievements in the continuum of care, if MDG 4 and 5 are to be achieved by the end of 2015. In addition to increased investments to develop a more responsive health system, particularly at community and primary care levels, the findings call for refined strategies and innovative thinking for better understanding of user behaviors, needs, and preferences, to improve maternal, newborn, and child health.
